# The Application of 3D Imaging as an Appropriate Method of Wildlife Craniometry: Evaluation of Accuracy and Measurement Efficiency

**DOI:** 10.3390/ani12233256

**Published:** 2022-11-23

**Authors:** Klára Košinová, Jiří Turek, Jan Cukor, Rostislav Linda, Martin Häckel, Vlastimil Hart

**Affiliations:** 1 Department of Game Management and Wildlife Biology, Faculty of Forestry and Wood Sciences, Czech University of Life Sciences Prague, Kamýcká 129, 165 00 Prague, Czech Republic; 2 Forestry & Game Management Research Institute, Strnady 136, 252 02 Jíloviště, Czech Republic

**Keywords:** 3D scanner, geometric morphometric, CT, reproducibility of results, antlers, mandible

## Abstract

**Simple Summary:**

Modern 3D imaging methods offer many scientific disciplines the opportunity to take their results to the next level. Using standard measurement methods, a CT scanner and a 3D scanner, we have established a methodology for using these devices for craniometric measurements. Craniometry is an important means of obtaining information on population quality and its long-term evolution, not only in wildlife species. In the case of these measurements, an important aspect is the accuracy and time efficiency of the methods used. It has been shown that 3D imaging technologies are able to obtain high quality data in comparable time to standard methods. A comparison of methods has proven that both the CT scanner and the 3D scanner can achieve accurate values, measure a larger range of variables, and store digital copies of the object for archiving or future research.

**Abstract:**

The suitability of CT and 3D scanners for craniometric proposes was tested using digital calipers when determining linear measurements, and a measuring cylinder was used for the accuracy of 3D printing of deer antlers obtained by the CT and 3D scanners. The resolution of digitized objects from a 3D scanner ranged from 0.008 mm to 0.122 mm. For mandibular dimensions, a positive deviation (*p* < 0.01) from the primary control measurement was recorded. The average antler volume measured with the cylinder was 60.47 cm^3^ at the first measurement, in the case of the CT scanner 61.62 cm^3^ and for the 3D scanner 64.76 cm^3^—both technologies exhibit a positive deviation from the primary measurement. Precise sensing and measurements can be used to evaluate the quality and evolution of wildlife populations, create digital museum collections, or to examine in detail certain traits such as antler and horn development or dentition.

## 1. Introduction

In the medical field, 3D imaging technologies are widely used, as well as computed tomography (hereafter referred to as CT) and magnetic resonance imaging (MRI), which allow for the creation of 3D images [1,2]. Because of the development of 3D technologies in recent years, devices that deliver accurate images of surfaces are increasingly developed and used, in addition to being affordable and often mobile [3,4,5,6]. These technologies are used in many branches of medicine; for example, for prosthesis creation, plastic surgery, orthodontics, or anthropometry [2,7,8,9,10].

However, these advanced technologies can be applied in more fields, not only healthcare. Object digitization offers additional possibilities for the detection of morphometric data. In recent years, there have been significant developments in a number of technologies for digitizing objects; at present, they are predominately used in archeology but are also used in veterinary and human medicine [11,12,13]. Photogrammetry has been used primarily for geographical measurements, especially for spatial planning, geomorphological analysis, or cartography [14,15,16,17]. Because of the rapid development of 3D imaging technology, geometric morphometry is often used in bioarchaeology [18,19,20,21,22,23], and one of the applications is craniometric data measuring. The process of scanning and digitizing an object differs according to the device used. The speed and quality of scanning an object usually depends on its size and complexity; for reasonably complex objects, this means dozens of scans [24]. In some cases, especially for complicated objects, hundreds of images are required for an accurate virtual reconstruction [25]. Today, 3D digitization is becoming an increasingly popular method; different objects are scanned for different purposes, such as for anthropological research, and in evolutionary biology, forensic science, archaeology, etc. [25,26,27,28,29,30,31].

### CT Scanning

Another important technology that can be used for craniometry purposes are CT scanners. Multidetector computed tomography (MDCT), or helical/spiral CT scanning, is now well-known [26]. MDCT represents the “third generation of CT devices”, which use helical CT scanning [27]. MDCT uses tomographs of various degrees of technical development, usually defined by the number of detectors (2–320) [28]. The use of standard “macroCT” devices is limited by insufficient knowledge of imaging limits for animal model species or by setting exposure parameters and CT protocols [29]. They are mostly used for large and exotic animals, which seem to be a suitable subject concerning the technical principles of CT, especially because of the volumetric similarity to humans [30]. The application in forensic or terminal ballistics, where the first attempts to set up and optimize protocols for the detection of missile fragments in cadavers can be seen, is still less frequent [31]. As part of optimization, basic craniometric measurements were performed when comparing dental protocols in selected species of dog carcasses [32].

The basic technology applied in craniometry is the measurement of dimensions using a caliper [33]. Craniometric dimensions include the length and width of the skull, volume of the neurocranium, length of the nasal bones and palatine, the rows of teeth, the length, height and width of the mandible, antler dimensions, and other parts [34], for which modern technologies such as 3D and CT scanning could be used. Craniometric traits may indicate intraspecific differences between populations in different locations, the evolution of a species over time, or may be seen as an indicator of an individual’s physical condition or health development or development of separate subpopulations of wildlife [35,36,37,38,39,40,41,42,43,44,45,46,47,48].

Due to the increasing populations of wild game [48,49,50], the need for their monitoring is required. Craniometry provides required outputs, and with the use of appropriate technology, a clear picture of population trends can be obtained. Based on this, proper management of the species can be suggested. Craniometric methods also include the measurement of antlers; basic quantities include the length or area, but for some species, especially cervids (*Cervidae*), the volume of the antlers is also measured, which is determined based on methods recognized by the CIC (International Council for Game and Wildlife Conservation) [51].

Therefore, craniometry provides valuable data on populations and individuals of animal species. However, manual measurement is time-consuming, and data accuracy can also be problematic. It should be mentioned that the use of digital methods offers a much greater range of spatial analyses. The morphological study of species thus acquires new perspectives [52,53]. However, it is necessary to know the level of accuracy of the obtained digital parameters and how they were established (methodical algorithm) to ably determine the error rate of any selected measurement algorithm. At present, the scanning procedures, manuals, and protocols of individual applications on a specific (e.g., animal) object and procedures of subsequent comparison in determining the accuracy of the performed measurement are not commonly available [54].

This study aims to describe the possible utilization of technologies well known in the health sector for multidisciplinary purposes. The main interest focuses on the resolution of scanned objects and the subsequent accuracy of the measurement in comparison with basic methods, such as measuring with a caliper or determining the volume using a measuring cylinder. The partial aims are to (i) compare three volumetric and linear measurement methods; (ii) describe the methodology and procedure for digitizing objects of interest with a CT and a 3D scanner for craniometric purposes; (iii) determine the measuring accuracy of both technologies with the standard method performed by a digital caliper using a calibrated reference object and the measurement limits of individual instruments to find the most accurate method; and (iv) evaluate the time effectiveness of the applied methods in comparison to the standard methodology.

## 2. Material and Methods

### 2.1. Study Objects

Two types of sample objects were used for the CT and 3D scanner craniometry, each from a different widespread (common) ungulate game species in Europe. In the first case, the mandibles of 60 wild boar (*Sus scrofa*), 1–14 months of age, were used. The object was chosen because it is well-suited for the selected methodology. The 3D scanner requires the objects to have a simple shape and a low contrast without excessively light, dark, or even transparent parts. In the second case, 30 skulls of male roe deer (*Capreolus capreolus*) with antlers, 1–6 years old, were selected (scanning focused on the antlersThe antlers represent a very complex, high-contrasted object with various shape extremes and variability for each sample. Both parts of the object (skull, antlers) are bone tissue, i.e., suitable for CT scanning.

The studied roe deer skulls come from the hunting districts of Radlice and Bohumile in the Czech Republic, Central Bohemia. Bohumile is a hunting ground with an area of 2900 ha. The area is located at an average altitude of 425 m above sea level and consists predominately of beech forests. At the Radlice hunting ground, with an area of 800 ha and altitude of 329 m above sea level, beech and mixed forests prevail. The selected hunting districts represent standard Central-European environments. All subjects were hunted as part of standard game management by hunters with valid hunting licenses and permits required in the Czech Republic. The animals were not hunted for study purposes.

### 2.2. 3D Scanner Measuring

The first device used for analysis was a 3D scanner, the ATOS (Advanced Topometric Sensor) Compact Scan 12M Essential Line. ATOS is a mobile contactless device created from stereo CCD cameras with a 12Mpx resolution (resolution is static) and a fringe projector. The ATOS measuring system is certified for metrology and supplied with certification according to regulation VDINDE 2634, Part 3. The device is placed on a positionable stand that enables stable anchoring of the scanner required for operation. Given the size of both types of the objects, cameras with a measuring volume of 300 mm were selected. The device utilizes blue light that enables scanning regardless of lighting conditions. The scanner uses the Fringe Projection method, in which precise stripes are projected onto the surface using a laser, which becomes deformed based on the object’s shape. The ATOS system uses a triangulation procedure. In order to achieve complete digitization, the object must be scanned from various angles. ATOS converts these images into a common coordinate system. The resulting set of measurement data is an STL file, 3D coordinates of points, sections, contour lines, or output quality protocols. The results of digitization can be exported to a system for Reverse Engineering, which is able to quickly reconstruct a 3D CAD model. GOM Inspect allows you to evaluate and compare measured data files with the default CAD.

The system requires regular calibration depending on the scan frequency and operating time. In our case, the device was calibrated once a week with the help of a certified calibration panel designed for a volume of 300 mm. For normal operation, it is necessary to set the correct scanning position of the cameras, the distance of the sensor from the object, the room temperature, and the stability of the device. The temperature on the turntable is always set during the calibration of the device and is checked before use. The scanner is capable of operating in the range of 5–40 °C. The optimal temperature is, therefore, around 20 °C; in our case, the device was calibrated to a temperature of 23.5 °C. The optimal distance of the scanner from the object is 60 cm. During commissioning, the actual scanning is preceded by heating the device for approx. 32 min.

**Figure 1:**
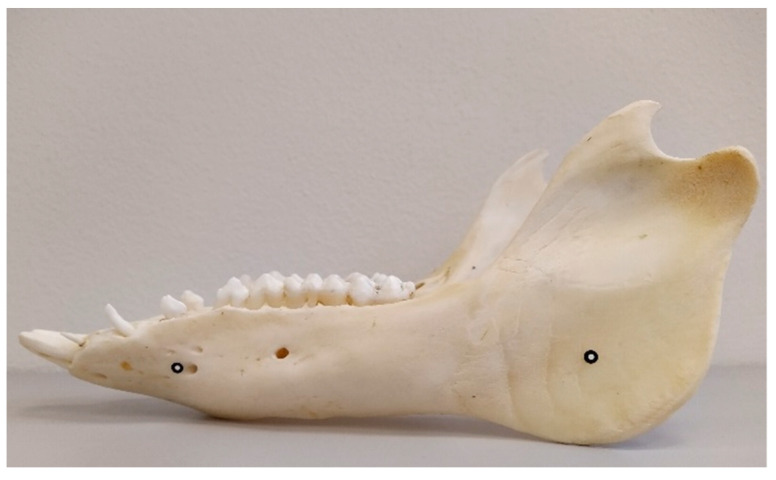
Location of reference points on the mandible of a wild boar (*Sus scrofa*)—left side, the same location on the opposite side because of the rotation and manipulation of the object.

**Figure 2:**
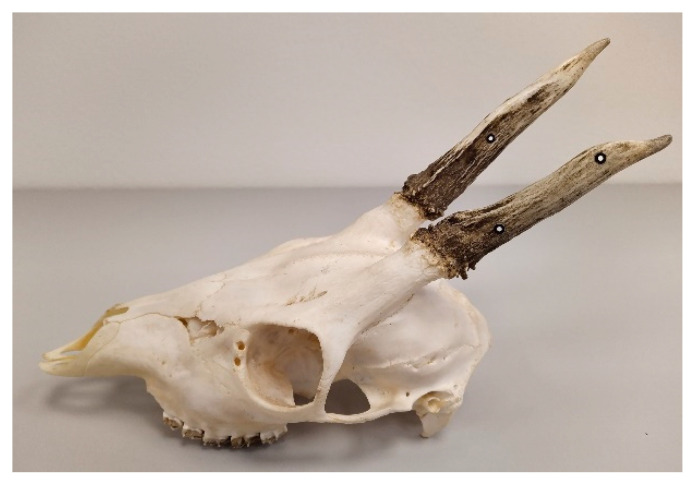
Location of reference points on roe deer antlers (*Capreolus capreolus*), the same location on the opposite side.

For both types of objects—mandibles and skulls with antlers—reference points of 1.5 mm were used (Table 1). Four reference points were placed on the mandible: two on each side of the mandible, one in the mental foramen (*foramen mentale*), and the other in the area of the angle (*angulus mandibulae*) (Figure 1). In the case of deer skulls with antlers, six reference points were placed symmetrically on each side. Their position was specified in relation to the articulation of the antlers. Two points were placed on the outer surface of the antler, one at the bottom, one at the top, and one point was placed on the inside of the antler (Figure 2). The reference points were also placed evenly on the turntable.

The object was then placed on an automatic turntable, Range Vision TL, with a diameter of 32 cm in the “basic position”. In the case of the mandible, a lateral view of the right side, and in the case of the roe deer, a frontal view of the nasal and frontal bone was created. The scanning of the object was carried out using the Gom Scan program. For both objects, the imaging was set to 8 images per 360° rotation of the turntable. The contrast of the object and reference points was set before scanning.

The mandible and the skull with antlers were scanned in two positions given the articulation of the object. Due to the high contrast between the skull and antlers, a chalk-based anti-reflective coating was used to scan the antlers. This spray unifies the color of the object, allows for faster and more accurate scanning, and is also easily removable. For more complicated objects, vaporizing anti-reflective spray can be applied. The automatic swivel pad ensures a constant scanning angle for each object. During scanning, automatic control of calibration, transformation, relative motion, and light change is performed with each image. For the antler scanning, 3–5 reference points located on the object were used to connect the measuring series. The resolution of the object scanned was recorded. After the conjunction of the measuring series, polygonization was performed at a standard level. The object was then exported. The output of the scanning is a digitized “mesh” object.

The entire sensing process was performed twice for each object to determine the sensing accuracy.

**Figure 3:**
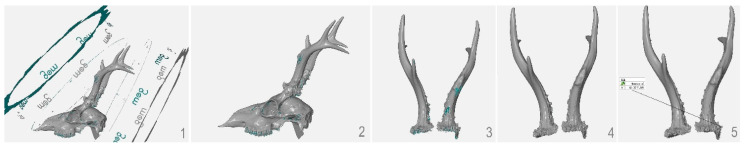
Process of object modification before measurement. 1. digitized object without modification (with unwanted parts of the turntable around the object); 2. mesh object after cleaning; 3. mesh object to be measured; 4. mesh object with filled holes, ready for measurement; 5. mesh object with the volume measurement results.

**Figure 4:**
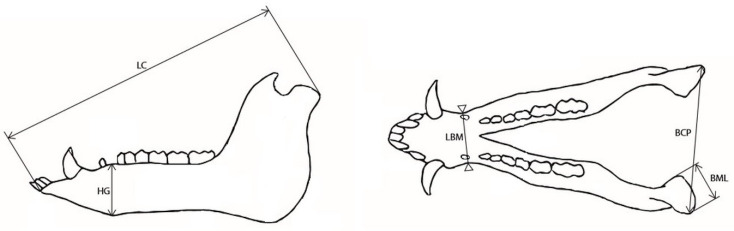
Dimensions measured on the mandible. Notes: LC—length of the cranium (condylobasal length), from the front part of *pars incisive* to anterior-most point of the *processus condylaris;* HG—height of the mandible, from the bottom of the *symphysis mandibulae* to the top of the *margo interalveolaris;* LBM—minimum breadth of the mandible; BCP—breadth of the mandible, between the borders of the medial and lateral points of the *caput mandibulae;* BML—breadth of the *caput mandibulae*.

The object was inspected using the GOM Inspect 2019 program, version 2.0.1. The primary functions of the software include the editing of a polygonal network, importing of the cloud of points and polygonal networks, sealing holes in the polygonal network by interpolation of the surrounding surface, smoothing, reducing, and thickening of a polygonal network, and triangle regularization. Basic modification of the object was based on removing randomly scanned elements from the vicinity of the object. In the creation of the antler mesh object, the antlers were first cut from the skull, and all residue of the antlers on the skull were removed, including the pedicles. In the case of a missing polygonal network, the openings were filled in so as not to disturb the natural shape of a part of the object and to prevent inaccuracies in the measurements (Table 2). Scanning was carried out in such a way as to avoid unnecessary hole formation. In the case of complex objects, it is not possible to completely prevent the formation of holes, but only to keep it to the necessary minimum by taking more images of the object in more positions. The object was scanned so that the maximum size of the unscanned area did not exceed 5 mm. Within this space, there were few holes predominately on the underside of the antler, sitting on the skull where it is not possible to scan the object (Figure 3). The dimensions measured on the mandible were chosen, from the previous experience, that best represent the development of the subject and reflect possible sexual dimorphism (Figure 4).

### 2.3. CT Scanner Measuring

A computed tomography scanner from Siemens, a Somatom Scope Power, was used as the second device. This is a multidetector computed tomography scanner (hereinafter referred to as MDCT) with 16 sections; in one rotation, it yields 16 sections of the scanned object. With the fastest pitch factor of 1.5, it can handle a rotation of 0.5 s and display the smallest section thickness of 0.6 mm, which helps to display relatively small anatomical structures and significantly speed up the examination. The gantry has an aperture diameter of 70 cm, but the field of view is 50 cm, which is identical to the reconstruction FoV (field of view). The data obtained from the slices are converted into a 1024 × 1024 matrix, whereas the reconstruction matrix is 512 × 512 pixels. The monitor resolution on which the scan output is displayed is 1280 × 1024 pix. The Somatom Scope Power device has extended input parameters and the voltage and the current of the X-ray here are 130 kV at 345 mAs (milliampere per second). A table with a maximum permissible load capacity of 210 kg cooperates with the CT, which makes it possible to examine even larger objects or specimens.

The position and centering of the specimen in the isocenter are important for setting up the specimen for scanning. In the case of a skull, it is necessary to center the quality of the image so that the resulting virtual representation and sections, axial and reconstructed, sagittal and coronal, are exactly in the selected plane. The horizontal centering corresponded to the level of approximately the external auditory canal (*meatus acusticus externus*). It was scanned in a position similar to a person lying on their back, i.e., almost in the frontal plane, which is suitable for perpendicular centering on antler coronets because of other circumstances and the purpose of use (measurement of antler volume was performed with a similar methodology as trophy evaluation in a measuring cylinder). In some cases, the position was reversed, meaning that the splanchnocranium was pointing down. This position was chosen in cases where it was necessary to align the sections of the coronets in a plane perpendicular to the growth axis of the antler.

**Figure 5:**
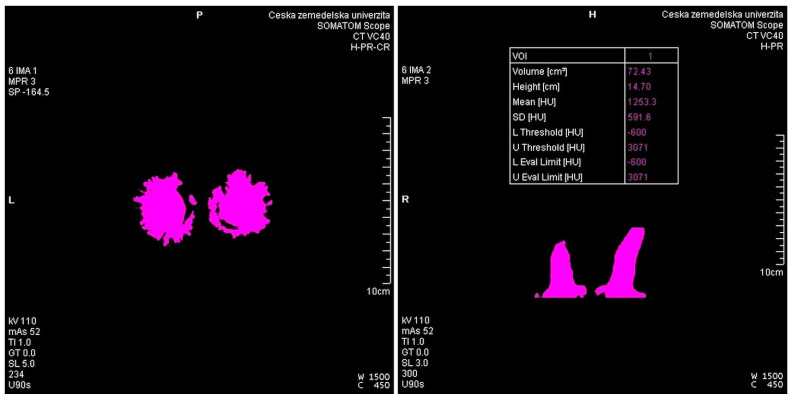
Measuring antler volume with a CT scanner.

The creation of the scanning protocol was inspired by the human default protocol for a child’s head, with only slightly adjusted exposure parameters—added mAs (milliampere-second) compared to the topogram-controlled CARE dose 4D (a specific system for current modulation of radiation, which is used to reduce the radiation load) automatic system, which is able to add mAs in larger areas for a better image quality because of temporal resolution while reducing the radiation dose in areas where intense “radiation” is not needed. The effective value of 180 mAs was never exceeded for a deer skull. For scanning and reconstruction parameters, the following parameters were the most important for the research: an “acquisition” section thickness of 2.0 mm (tolerance ± 0.5 mm) and a reconstruction section of 1.0 mm (tolerance ± 50%). The collimation of the detectors was 16 × 0.6 mm, i.e., the lowest possible and the recon increment was 0.5 mm. The kernel selected was U90 ultrasharp to enhance the density interfaces, i.e., the accuracy of the edges and contours to increase the accuracy of the measurement. For the virtual reconstruction, the Syngo CT VC40 program was used. A standard bone window was selected, which sufficiently displays both the antlers and the teeth in our case. The bone window is clearly defined with brightness and contrast. The width of the window was 3000 HU (Hounsfield units), and the window center was 400 HU. The acquisition FoV 1 corresponded to the whole object, i.e., the skull including antlers, with the FoV2 corresponding to the second reconstruction carefully defined only in the center of the coronets—as close as possible to the perpendicular line to the axis of their growth up to the entire antler. A pitch compromise of 1.0 between speed and image quality proved to be a very suitable solution (Table 3). From the acquisition, an MPR was created—a multiplanar reconstruction and a measured object in a bone window using the “volume rendering” technique, i.e., VRT. The measurements were performed in a bone window and on an aligned anatomical plane, always accounting for the one that needed to be measured, i.e., correlated with the same section. The measurements of antler volumes were performed in the Volume SW Siemens Syngo application, and they were considerably simplified by the previous methodology of creating the 2nd reconstruction and the selected FoV, when a part of the skull was already cut off, and only the volume of the antlers was measured (Figure 5).

The same exposure and reconstruction parameters were used to measure the mandible. The same FoV was used for the 2nd reconstruction and centering, as well as storage in the isocenter. The laser was centered on the gnathion (i.e., at the junction of the two parts of the mandible), and the height centering thus corresponded to approximately half the height of the mandible. The entire scanning process was performed twice for each object to determine the sensing accuracy.

### 2.4. Primary Measurement

#### 2.4.1. Digital Caliper

Mandibular quantities were measured using a digital caliper Kinex 6040-02-300. Calibration was performed before each measurement. The measurement accuracy specified by the manufacturer varies between 0.02 mm and 0.04 mm, depending on the object measured and the measurer. Measurements were performed on all mandibles in two repetitions, i.e., all dimensions on each mandible were measured four times. The intention was to detect the error made by the caliper and at the same time, to eliminate it for the determination of the average values of each dimension.

#### 2.4.2. Measuring Cylinder

The volume of the antlers was measured by the method determined by CIC [51]. This method is based on the hydrostatic law of determining the weight of water displaced by the antlers. The measurement is performed using pharmacy scales with a capacity of up to 1000 g and a sensitivity of 1 g—Kern PCB1000, in our study. First, the trophy is weighed in the air to determine the gross weight. In the second measurement, the antlers were immersed in water in a graduated cylinder, with a volume of 10 L, up to the lower edge of the coronets so that they did not touch the walls or bottom of the vessel. The difference between the values of the first and second measurements indicated the weight of water expelled by the antlers in grams. One gram of displaced water represented 1 cm^3^ of the antler volume [55] (The volume measurement process was performed twice to determine the accuracy of the method).

#### 2.4.3. Reference Object

For calibration purposes, a silicone object (KRO) was created using a 3D printer. This object was subsequently adjusted on a milling machine and handed over to the CMI (Czech Metrology Institute) for calibration. The object was created to suit both technologies, mainly in terms of material and surface.

Based on CMI recommendations, roe deer antlers were scanned and printed from ASA (acrylonitrile-styrene-acrylic) and subsequently re-measured for further comparison of measurement accuracy. Antlers represent a complex object that can show scanning errors, unlike a simple KRO.

#### 2.4.4. Statistical Analyses

In all cases, a paired *t*-test was used to determine the significance of deviations of the scanner and CT measurements from the reference caliper measurement. The dependence of the measurement accuracy of the antler volume measurement determined by scanning and CT measurement on the actual antler volume (measured by the reference method) was analyzed by linear regression. All calculations were performed in software R [56]. The significance level chosen for all statistical tests was α = 0.05.

The initial evaluation of differences between repeated measurements of identical samples was evaluated using 95% confidence intervals computed using *t*-distribution.

## 3. Results

**Figure 6:**
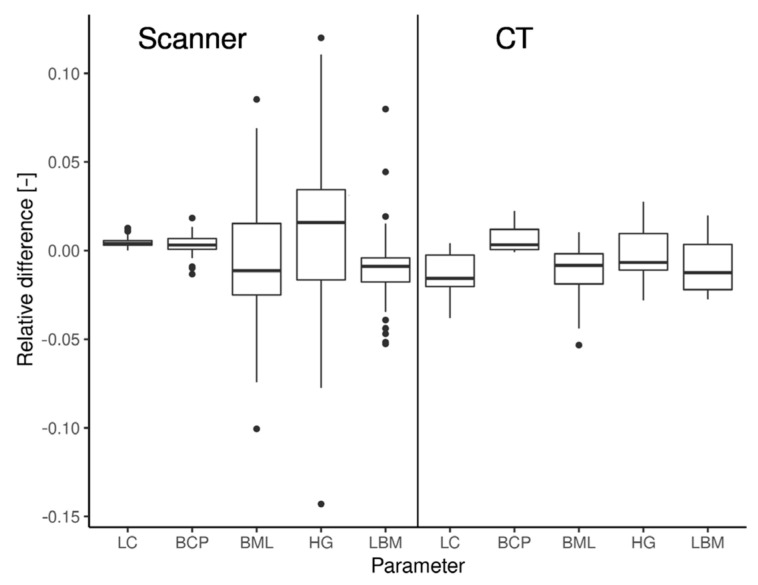
Box plot of relative deviations of measured parameters (X) by selected methods (see text in upper part). The deviances were standardized by hand measurement using the caliper.

For the first analysis, we computed absolute and relative deviations between both measurements with the caliper and the measuring cylinder. The mean absolute difference between caliper measurements in the case of the LC parameter is 1.27 cm (95% CI 0.79–1.75), which accounts for a relative difference of 0.39–0.86%. Low deviations were also observed in the case of the HG parameter—mean absolute difference = 0.67, 95% CI = (0.39–0.96), which accounts for a relative difference of 1.2–2.9%. For measurements in the measuring cylinder, a mean deviation of 4.3 cm^2^ (95% CI 3.83–4.77) was observed, which accounts for a relative difference of 6.2–7.7% (Figure 6).

The deviation of the 3D scanner and CT measurements of five selected mandible parameters were evaluated by analyzing the absolute and actual deviations from the measurements collected using a caliper. The highest absolute deviation (i.e., the lowest observed deviations computed as $\left|m_{m}-m_{c}\right|$, where m_m_ stands for measurement by one of the selected methods and m_c_ for primary measurement by caliper) was in the measurement using the 3D scanner of the parameter BCP (mean = 0.42 mm), followed by LBM (0.50 mm), BML (0.54 mm), LC (0.87 mm), and HG (1.04 mm). When measured by the CT method, the BML parameter showed the lowest absolute deviation (mean = 0.32 mm), followed by HG (0.46 mm), LBM (0.51 mm), BCP (0.58 mm), and LC (3.00 mm).

When comparing the actual deviations of measurements derived from 3D or CT scans with manual caliper measurements (computed as $\left|m_{m}-m_{c}\right|$), we found significant differences for three of the five parameters in the 3D scanner measurements and two of the five parameters in CT measurements. The results of the analysis of the actual deviations of measurements are depicted in Table 4.

*p*-values were provided for the null hypothesis, which means the actual difference of the selected parameter by the relevant method (CT/scanner) is equal to zero.

In the case of the LC parameter, a significantly positive deviation of the scanner measurement from the primary measurement was observed. This contrasts with the results from the CT method, where a significant negative deviation was observed for this parameter (the CT method, which significantly underestimated the length of this parameter). For the BML and HG parameters, no significant deviations were found in either case. The LBM parameter was significantly underestimated in the case of scanner measurements; no significant deviation was observed in the case of CT measurements (Table 4).

The accuracy of the digital caliper, 3D scanner, and CT measurements based on two independent measurements of the LC and HG dimensions was compared. The difference was insignificant, although this method recorded the largest average difference between the first and second measurements of 1.27 mm for LC and 0.67 mm for HG. Conversely, the lowest mean difference was recorded when using the 3D scanner: 0.15 mm for LC and 0.05 mm for HG (Table 5).

**Figure 7:**
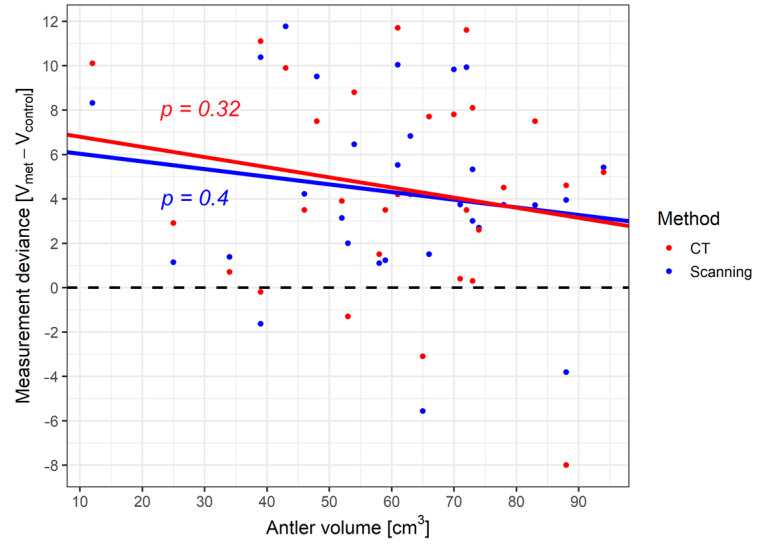
Dependence of deviations of antler volume measurements by the investigated methods on the actual antler volume. R-squared values: Scanner—0.025, CT—0.036.

Analysis of the dependence of deviations in the measurement of antler volume using the tested methods on the actual antler volume showed an insignificant trend (CT, *p* = 0.317; 3D scanner, *p* = 0.405) for the scanner and CT measurement method. In our experiment, both technologies slightly overestimate the volume of the antlers, independent of their actual volume. For the measurements derived from the CT scan, the average deviation from the reference measurement is 4.49 cm^3^ (95% confidence interval 2.77–6.209). The data from the 3D scanner shows a positive deviation of an average of 4.293 cm^3^ (95% confidence interval 2.738–5.847). The largest deviation was recorded for antlers with a smaller volume, with the deviation decreasing with increasing volume (Figure 7).

The displayed *p*-values apply to the null hypothesis of a slope of the linear regression line equal to 0.

The second measurement was taken to determine the accuracy of the volume measurements. The graduated cylinder measurement showed the greatest difference between the first and second measurement, averaging 4.30 cm^3^. The CT and 3D scanner showed the least difference in the measured values, with the 3D scanner yielding the more accurate measurement with an average difference of 0.84 cm^3^ between the first and second measurements (Table 6).

**Figure 8:**
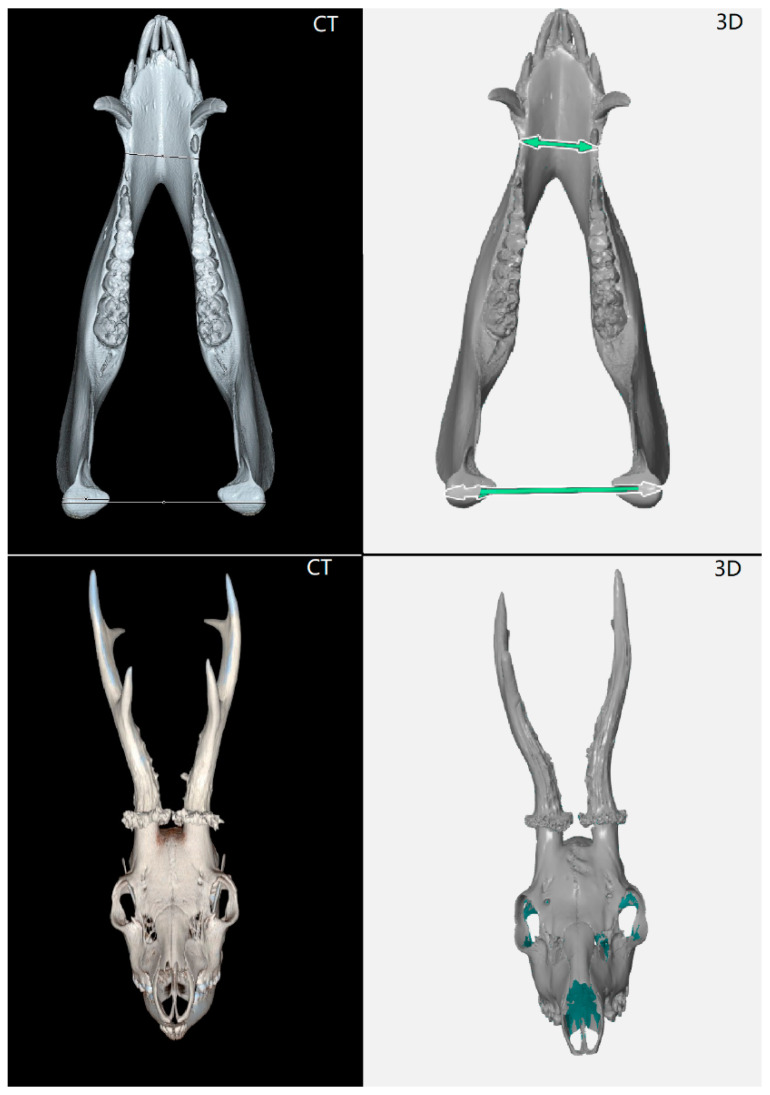
Comparison of imaging capabilities of a CT/3D scanner.

**Figure 9:**
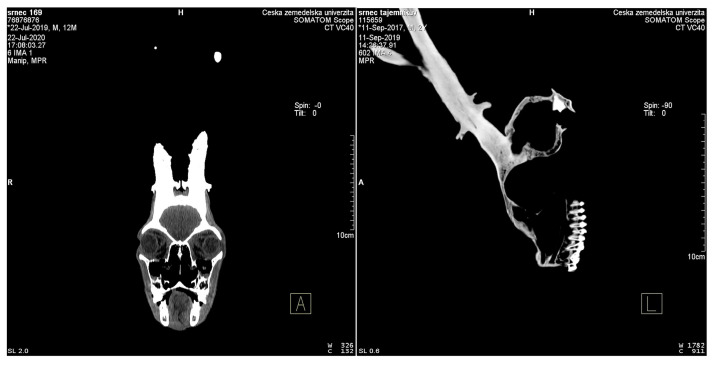
CT scanner—an internal image of a roe deer skull with visible tooth roots and antler structure.

**Figure 10:**
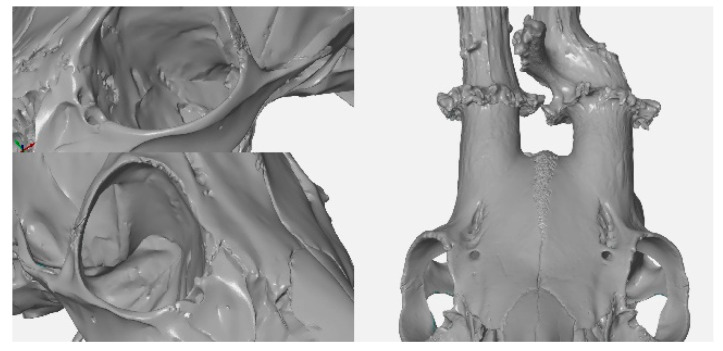
3D scanner—a detailed view of external structures, including view of the *fossa lacrimalis externa*, and detail of cranial vault sutures.

Due to the volatility of measurement accuracy and differences in the use of the primary method (digital caliper), a comparison of the measurement accuracy of the 3D and CT scanners was performed with a reference object, which was calibrated by the CMI. These measurements and subsequent comparisons showed negligible differences in accuracy, with the maximum deviation from the reference measurement being 0.1355 mm in the case of the length of C measured with a CT scanner. Both the 3D and CT scanner showed a negative deviation of 0.00855–0.1355 mm for the length measurements (Table 7). The volume measurement showed a positive deviation of 0.062872 cm^3^ for the 3D scanner and a negative deviation of 0.426 cm^3^ for the CT scanner (Table 8). A subsequent print of a duplicate of the antlers showed a difference of 1.35 cm^3^ between the measurement by the CT scanner and the 3D scanner (Table 8). A comparison of the two technologies showed that it is possible to obtain similar outputs in a similar time (Table 9). The main difference is in the resolution: it is 0.01 mm for the 3D scanner, and 0.6 mm for the CT scanner. The 3D scanner is more suitable for scanning the surface of the object, whereas contrarily, the CT scanner has the considerable advantage of scanning the interior of the object (Table 9, Figure 8, Figure 9 and Figure 10). The CT scanner offers the possibility of measuring the distance between points, angle, volume of the entire object, volume of internal spaces, volume of partial parts, density, and tissue structure. In the case of the 3D scanner, the main difference of the measurable dimensions is that it cannot scan (and thus, evaluate) the internal spaces and then measure the density.

## 4. Discussion

Craniometry based on direct measurement, whether using a caliper or digitized scales, is taking a back seat and modern technologies are increasingly becoming favored. Measurements of 2D dimensions, such as of length or width, have proven very useful and have become common in human or veterinary cardiology and angiography (e.g., in measuring stenoses of important arteries for use in therapy) [33], as well as in dentistry and implantology [57]. Even today, many craniometric studies use a digital caliper with an accuracy ranging from 0.01 to 0.5 mm as the primary measuring method [41,47,58,59,60,61,62]. In the case of a 3D scanner, when reconstructing the roe deer antlers [63], rotator cuff [64], and vertebrae [65], the accuracy ranges from 0.01 to 0.1 mm; for human skulls [66], the tissue equivalent phantom [67] and elbow joint [68], is 0.33–1.2 mm in a CT scanner. The resolution found in our measurements for the 3D scanner ranged from 0.008 to 0.122 mm, and the accuracy ranged from 0.01 to 0.29 mm. The accuracy of the CT scanner ranged from 0.14 to 1.45 mm; therefore, the 3D scanner seems to be a more accurate device. The set up and the calibration of the device may affect the resolution, but the data obtained correspond to the technical specifications stated by the manufacturer. The great advantage of scanners, both 3D and CT, is that the measurement and scanning of the object are performed in a non-contact manner. Thus, the measuring unit does not come into direct contact with the measured object, which reduces difficulties, limitations, and the possibility of error. This increases measurement accuracy and reduces the risk of potential damage to the measured object held in the hand of the person measuring it [69,70,71,72]. The accuracy range is significantly higher than that of hand-held 3D scanners [63,64]. In addition, the accuracy of multi-row CT scanners is at a submillimeter level and determined by technical principles, mainly the width of the detectors, which is proportional to the voxel size, usually from 0.4 mm and above. Measurement with these scanners is thus a significant benefit, first in dentistry (especially in more complex reconstructive procedures) and now in implantology (e.g., modeling of artificial heart valves to measure, etc.) [57].

The positive deviation of the LC and BCP parameters measured by the 3D device is apparently the result of the inaccuracy of measurements when using digital calipers. These are the longest parameters measured on the mandible. These parameters are the measurements of the greatest distance on the mandible—any inaccuracy will necessarily manifest itself primarily in them. This is also confirmed by the insignificant deviation found for the BML and HG parameters (smallest measured dimensions, Table 1). The negative deviation of the LBM parameter is caused by inaccurate measurements using a digital caliper and apparently, the measuring device itself. Accurate determinations of craniometric points for LBM measurements are problematic and burdened with a significant degree of subjective error; with calipers, there are significant deviations, whereas using 3D scans, it is possible to determine the starting points for measurements more accurately. The main reason for the negative deviation of the LC parameter measured by MDCT may be the difficulty of detecting the starting points of the measurement in a given plane. One way to solve the problem is additional post-processing, with the creation of additional sections that accurately capture both points, but such a solution seems quite uneconomical with respect to time, and the outcome is uncertain.

In the case of measuring antler volumes, CT and 3D devices showed very similar results. Compared to the reference measurement performed by the measuring cylinder method, a positive deviation was recorded for most antlers. The largest deviation was recorded for antlers of smaller volumes, which indicates a direct relationship between the measurement accuracy and the volume of the antlers. The main advantage of both technologies is the possibility of precise delimitation of the part of the object whose volume we want to determine. This difference is most noticeable in geometrically complex antlers, or antlers that are on the pedicles at a very slight angle; such antlers are difficult to measure with a measuring cylinder—the level of the fluid in the cylinder is always horizontal, and the antler with the skull often cannot be inserted perpendicularly to the horizontal plane. The printing of a duplicate of the antlers showed a similar trend, which was recorded for most measured volumes of antlers; the CT scanner shows slightly lower measured values than the 3D scanner, which is probably due to the somewhat more complicated measurement of volume by the CT scanner and an error of the measuring device.

When using CT, the original intention was to perform all measurements in one (axial) section showing the plane and the two farthest points clearly, which turned out to be relatively time-consuming and difficult to implement. Such a methodology does not guarantee that the selected section will display the correct starting and end points of the measurement precisely. Even with careful centering of the object, there will be either a slight deviation, which the human eye is unable to recognize, or an error due to overlooking the natural lateral asymmetry of “bilaterally symmetrical animals (Bilateria)”, i.e., mammalian skulls. According to the device specification, deviations of positional lasers for all measuring planes in the value of ±2 mm are also given. Concerning similar research [66], we chose measurements using 3D VRT reconstruction, which we compared with axial sections on 10 samples (measured 10 times on each on different planes). On the one hand, the measurement helped us to speed up the work, and on the other, we evaluated the resulting deviation (amounting to 0.5%) as normally acceptable and comparable with the results of other authors [73,74,75]. The advantage of MDCT for similar contrasting objects (mandible, antlers) is the relatively easy distinguishability of HU differences, but there still remains the need to precisely set the imaging limits (in HU). For antlers, the main challenge was to capture their cancellous part with a relatively high gas (air) content, and the need to include the gas density within the limits. The advantage of MDCT, although it does not have such a high resolution of details of the examined objects, is the non-destructive nature of the imaging methodology, especially the possibility of 3D reconstructions.

The 3D scanner Atos Compact Scan is more appropriate and accurate for measuring smaller objects whose size is not too far from the center point in any direction. High-contrasted objects with glossy surfaces appear to be problematic. This problem can be solved by using (removable) anti-reflective sprays to accurately digitize even very dark as well as shiny objects without significantly affecting the accuracy of the measurements [73]. Contrastingly, CT Siemens, Somatom Scope, work more accurately on larger objects. This is mainly due to the much lower output resolution (compared to the 3D device) when the output voxel is significantly larger, and thanks to the defined layer width, it is possible that smaller objects will be captured at the interface of the two detectors. The resulting accuracy of measurements, verified by a calibrated reference object, shows a near equivalence of accuracy in both methods, which is significantly higher compared to the methods used so far [63,64,65,66,67,68,76,77]. Moreover, all methods have proven to be almost equally time-consuming, which again leads us to choose a more accurate and replicable method.

## 5. Conclusions

Our proposed methodologies show the considerable potential of both technologies used; each of them brings specific outputs with a wide range of applications. The established methodology is substantially effective and demonstrates the correct use of instruments for craniometry purposes. Detailed settings and the method of treatment of the digitized objects show effective possibilities for scanning the skulls of animals and subsequent measurements of quantifiable values. With regard to the comparison of measurement accuracy with a calibrated reference object, it is also possible to state that both technologies have high measurement accuracy, which ensures both obtain accurate results in a very short time, and the possibility of their subsequent sharing and use for a wide range of research. The recorded deviations were negligible in the case of KRO and indicate the reliability of the methods used. Although differences in dimensional measurements may seem insignificant, these values are crucial for many studies.

Both technologies allow for not only measuring standard craniometric dimensions, but through them, we can also determine quantities such as the volume of individual segments of the object, angles, lengths of curves, tissue density, and dimensions of internal spaces. Both the 3D and CT scanners facilitate archiving and digitization across various fields. A future scenario could include the creation or fulfillment of existing digital libraries of 3D scans for the sharing and further use of data by scientists globally. Only a combination of both technologies and created methodologies can achieve comprehensive and quality outputs.

## Figures and Tables

**Table 1:** Object scanning process.

	Camera Volume	Reference Points	Number of Images	Number of Positions	Additional Preparation	Number of Sensing Processes
Mandible	300 mm	4	8	1	none	2
Skull	300 mm	4–7	8	2	anti-reflective spray	2

**Table 2:** Object postprocessing.

Object	Polygonization	Program	Basic Modification	Object Correction	Measurement
Mandible	Standard level	GOM Inspect 2019	removing randomly scanned elements	sealing holes in the polygonal network	Measuring the distance between two points
Skull	Standard level	GOM Inspect 2019	removing randomly scanned elements	sealing holes in the polygonal network, cutting antlers from the skull	Measuring the mesh volume

**Table 3:** CT protocol and measurements.

	Scanning Protocol	Thickness of the “Acquisition” Section (mm)	Reconstruction Section (mm)	Kernel	Reconstruction	Measuring
Mandible	Child’s head	2	1	U90	FoV	aligned anatomical plane
Skull	Child’s head	2	1	U90	FoV2	Volume SW Siemens Syngo application

**Table 4:** Results of statistical testing of measurement deviations using both methods from primary measurements for all (60 samples) selected mandible variables.

Variable	Scanner			CT		
	Average Deviation (mm)	*p*-Value	Mean Relative Deviation (%)	Average Deviation (mm)	*p*-Value	Mean Relative Deviation (%)
LC	0.873	**<0.001**	0.48	−2.683	**0.014**	1.57
BCP	0.285	**<0.001**	0.49	0.543	**0.02**	0.73
BML	−0.069	0.42	2.83	−0.222	0.10	1.76
HG	0.293	0.08	3.48	−0.143	0.47	1.37
LBM	−0.342	**<0.001**	1.64	−0.298	0.10	1.55

Notes: LC—length of the cranium (condylobasal length), from the front part of the *pars incisive* to anterior-most point of the *processus condylaris*; HG—height of the mandible, from the bottom of the *symphysis mandibulae* to the top of the *margo interalveolaris*; LBM—least breadth of the mandible; BCP—breadth of the mandible, between the borders of the medial and lateral points of the *caput mandibulae*; BML—breadth of the *caput mandibulae*.

**Table 5:** Differences in the accuracy of the methods used for distance measuring of all wild boar mandibles.

Method	Para-Meter	Mean 1st Measurement/Scanning (mm)	Mean 2nd Measurement/Scanning (mm)	*p*-Value	Max Difference between 1st and 2nd Measurement/Scanning (mm)	Min Difference between 1st and 2nd Measurement/Scanning (mm)	Average Difference of 1st and 2nd Measurement/Scanning (mm)
Caliper	LC	202.94	203.06	0.996	2.28	0.22	1.27
	HG	33.50	33.50	0.999	1.63	0.24	0.67
3D scanner	LC	203.94	203.96	0.999	0.29	0.05	0.15
	HG	34.07	34.07	0.999	0.13	0.01	0.05
CT scanner	LC	207.26	207.20	0.998	0.98	0.18	0.44
	HG	33.30	33.12	0.958	1.45	0.14	0.57

**Table 6:** Difference in the accuracy of the methods used for volume measuring of all (30 samples) roe deer antlers.

Method	Mean 1st Measurement/Scanning (cm^3^)	Mean 2nd Measurement/Scanning (cm^3^)	*p*	Max Difference between 1st and 2nd Measurement/Scanning (cm^3^)	Min Difference between 1st and 2nd Measurement/Scanning (cm^3^)	Average Difference of 1st and 2nd Measurement (cm^3^)
Cylinder	60.47	62.83	0.646	7.00	2.00	4.30
3D scanner	64.76	64.87	0.982	3.27	0.02	0.84
CT scanner	61.62	64.96	0.493	6.00	0.80	3.33

**Table 7:** Comparison of the measurement accuracy of both technologies on a reference object calibrated by the CMI (Czech Metrology Institute).

	CMI	3D Scanner	CT Scanner
Length of side A (mm)	83.85485	83.729	83.7
Length of side B (mm)	83.83305	83.812	83.8
Length of side C (mm)	83.90355	83.895	83.8
Volume (cm^3^)	589.826	590.455	589.4

**Table 8:** Comparison of the measurement of antlers on the skull and the printed duplicate.

Volume of Antlers on the Skull (cm^3^)			Volume of the Printed Antler Duplicate (cm^3^)		
Measuring Cylinder	CT	3D Scanner	Before Printing According to GrabCAD	CT	3D Scanner
94.00	99.20	99.42	99.42	97.40	98.75

**Table 9:** Comparison of the limits of both technologies.

	Accuracy (mm)	Scanning Preparation (min)/Preparation	Scanning Time of Mandible/Antlers (min)	Post-Processing Time of Mandible/Antlers (min)	Scannability of Surface Limits	Scannability of Internal Spaces	Special Requirements
CT	0.6	3	2/3	3	Metal-based materials create artifacts	Dependence on the permeability of the outer material for X-ray and the overall size of the object, soft tissue detection in small animals	Radiation protection
3D	0.01	3	2/5	2/8	Feathers and fur, shiny high-contrast surfaces after the use of anti-reflective spray	Not possible	No special requirements
Digital caliper	0.02–0.04	0	0	5/10	Unrepeatable; only distance and volume	Not possible	No special requirements

## Data Availability

The data presented in this study are available on request from the corresponding author. The authors want to keep track of who is working with the data for possible collaboration.
